# Taming conformational heterogeneity in and with vibrational circular dichroism spectroscopy†
†Electronic supplementary information (ESI) available: Full description of the used genetic algorithm and cross-validation methods, additional raw and processed spectra and additional table with number of conformations at different levels of theory. See DOI: 10.1039/c9sc02866h


**DOI:** 10.1039/c9sc02866h

**Published:** 2019-07-09

**Authors:** Mark A. J. Koenis, Yiyin Xia, Sérgio R. Domingos, Lucas Visscher, Wybren Jan Buma, Valentin P. Nicu

**Affiliations:** a Van't Hoff Institute for Molecular Sciences , University of Amsterdam , Science Park 904 , 1098 XH Amsterdam , The Netherlands . Email: w.j.buma@uva.com; b Deutsches Elektronen-Synchrotron DESY , Notkestraße 85 , 22607 Hamburg , Germany; c Amsterdam Center for Multiscale Modeling , Division Theoretical Chemistry , Faculty of Sciences , Vrije Universiteit Amsterdam , De Boelelaan 1083 , 1081 HV Amsterdam , The Netherlands; d Radboud University , Institute for Molecules and Materials , FELIX Laboratory , Toernooiveld 7c , 6525 ED Nijmegen , The Netherlands; e Department of Environmental Science, Physics, Physical Education and Sport , Lucian Blaga University of Sibiu , loan Ratiu Street Nr. 7-9 , 550012 Sibiu , Romania . Email: v.p.nicu@gmail.com

## Abstract

The flexibility of a molecule has important consequences on its function and application. Vibrational Circular Dichroism (VCD) is intrinsically an excellent experimental technique to get a hold on this flexibility as it is highly sensitive to key conformational details and able to distinguish rapidly interconverting conformers. One of the major challenges in analyzing the spectra by comparison to theoretical predictions is the uncertainty in the computed energies of the multitude of conformations. This uncertainty also affects the reliability of the stereochemical assignment it is normally used for. We present here a novel approach that explicitly takes the energy uncertainties into account in a genetic algorithm based method that fits calculated to the experimental spectra. We show that this approach leads to significant improvements over previously used methodologies. Importantly, statistical validation studies provide quantitative measures for the reliability of relevant parameters used such as the energy uncertainty and the extent to which conformational heterogeneity can be determined. Similarly, quantitative measures can be obtained for the possibility that the flexibility that is introduced in the fit might lead to an incorrect assignment of the stereochemistry. These results break new ground for different techniques based on VCD to elucidate conformational flexibility.

## Abstract 2

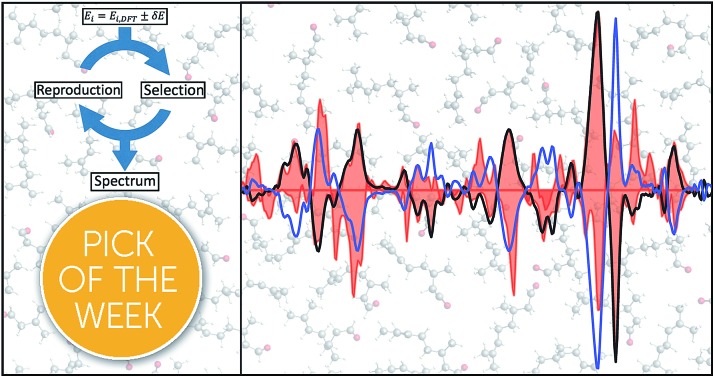
Genetic fitting algorithm accounting for the uncertainty in computed energies allows a significantly more reliable assignment of stereochemistry and conformational heterogeneity of chiral compounds using vibrational circular dichroism.

## 1 Introduction

The interplay between structure and function of a (bio)molecule is intrinsically governed by conformational flexibility. Revealing the intricacies of the complex conformational landscapes of flexible molecules is therefore a fundamental target of advanced spectroscopic techniques, not only for structural determination, but also for defining routes for conformational relaxation and internal dynamics. Vibrational circular dichroism (VCD)^1^–^3^ – the differential absorption between left- and right-circularly polarized light in the vibrational infrared frequency domain – has shown its unique strength for determining the absolute configuration of chiral molecules under conditions in which these compounds are actually employed. At the same time, it has shown to be a powerful technique to provide information on the thermal distribution of conformations present at room temperature in the liquid state. The reason for this is that the differential nature of the VCD spectrum leads to positive and negative bands with signs and strengths that are more sensitive to the finer details of the conformational structure than the strength of bands in the vibrational absorption (VA) spectrum.^4^

To interpret experimentally obtained VCD spectra, Density Functional Theory (DFT) calculations are commonly used. For cases in which the molecule has several conformations that are thermally accessible – as will be the case for the flexible systems on which we focus here, one generally uses a Boltzmann-weighting of the spectra of the individual conformations to construct an ‘experimental’ spectrum that is then compared with the experimentally obtained spectrum. However, inherent to the calculations is an uncertainty in energy which depends on molecular structure and level of theory, but as a rule of thumb is taken to be at least of the order of 1–2 kcal mol^–1^.^5^–^8^ Such an uncertainty has serious implications because it directly affects the weighted averaging of the spectra of the various conformations. This is particularly important if, as is often observed, the spectra of different conformations display in the same frequency region features with opposing signs, similar to what would be observed if this region would be compared for the two enantiomers of that compound.^9^ As a result, calculations at different levels leading to different weightings of conformations may in the end very well lead to opposite conclusions on the absolute configuration of the compound of interest. The uncertainty in energy thus not only impedes an unambiguous comparison between experiment and theory, but also challenges the proclaimed and by industry required ∼100% confidence in enantiomeric specification.

**Fig. 1:**
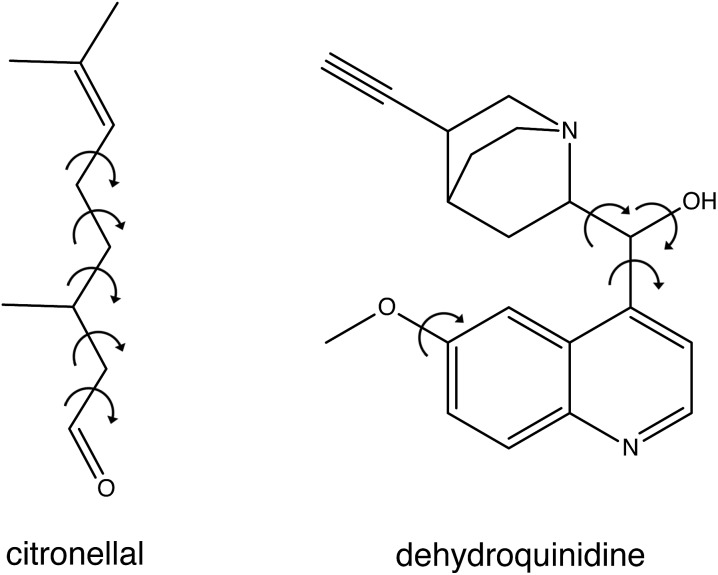
Chemical structure of citronellal and dehydroquinidine with the arrows showing the free rotational bonds that are the main source for their structural flexibility.

In the present studies we have developed a novel approach to compare experimental spectra with theoretically predicted ones. To this purpose we fit the experimental spectrum with an energy-weighted sum of spectra predicted for the various conformations, but explicitly take the uncertainty in the calculated energies into account. We demonstrate the strength and advantages of this approach by applying it to the VA and VCD spectra of citronellal and dehydroquinidine (see Fig. 1). Citronellal is a chiral acyclic monoterpene and a versatile precursor in the biosynthesis of isopulegol isomers. As an odorant molecule with known uses as insect repellent and within the cosmetics industry, citronellal is a customary target of studies that focus on revealing binding efficiencies and interactions with olfactory receptors.^10^–^12^ Its linear structural arrangement, consisting of five carbon–carbon single bonds, gives rise to a complex conformational phase space with a plethora of structures that can be accessed at room temperature of which 15 were identified in recent broadband rotational spectroscopic studies under molecular beam conditions.^13^ However, the real world in which these molecules are actually applied is one in which molecules are at room temperature and under non-isolated conditions. As a result, they are able to convert from one conformation to another, and experience interactions with their environment. Such intermolecular interactions can have major effects on conformational energies and structures, and previous understandings of the potential energy landscape provided by gas-phase studies may thus need considerable revision. Therefore, a more complete understanding of the structural aspects of these flexible molecules and how this reflects on their functionality must come from solution phase studies.

Dehydroquinidine belongs to the class of cinchona alkaloids, compounds that have found widespread use in asymmetric organocatalysis. The conformational landscape of these compounds has been extensively studied as it dominantly influences the outcome of an asymmetric reaction.^14^–^23^ Driven by apparent conflicting conclusions in other VCD studies, we investigated recently how structural dynamics affect the VCD spectra of these compounds,^24^ and in particular that of dehydroquinidine. Compared to citronellal, dehydroquinidine can be considered as a ‘worst-case’ test for our approach. Experimentally, the recorded VCD spectrum suffered from a non-optimal baseline since only one stereoisomer was available and the baseline therefore had to be obtained from measurements on the pure solvent. Theoretically, we found that calculated VCD spectra depend sensitively on the Boltzmann weights (BW) of the large number of conformations accessible at room temperature, but that these BWs varied considerably for calculations with different computational parameters. Furthermore, large-amplitude motions involving the OH group turned out to influence dramatically signal intensities in the OH-bending region of the spectrum, even to such an extent that only by taking explicitly structural dynamics into account an acceptable agreement between experiment and theory could be obtained.

Here, we will show that for both systems our approach works equally well and has key advantages. Utilizing conformational energies that mimic reality as much as possible, we have been able to investigate the conformational heterogeneity of citronellal in neat and solution phases and explore the potential energy surface of this flexible molecule in a strongly interacting environment. These studies demonstrate that for a prototypical flexible molecule such as citronellal our approach leads to an improvement in the agreement between experiment and theory and that it allows for a much more secure assignment of the absolute configuration. The same conclusions are drawn in the studies on dehydroquinidine, despite the drawbacks indicated above. What is in this case, however, particularly relevant is that we find that the increased fitting flexibility does not lead to different conclusions on the intrinsic physics of the system, that is, the effects of the structural dynamics cannot be reproduced by loosening the conformational energy restrictions. Importantly, we will show that measures can be derived to quantify the confidence level of the assignment of the absolute configuration. Such measures are necessary since the increased fitting flexibility could in principle lead to an incorrect stereochemical assignment. Finally, our fits lead to a predicted conformational distribution that can be probed independently by other experimental techniques.

## 2 Methods

The vibrational absorption and VCD spectra have been recorded using a Biotools Chiral-2X spectrometer and a 75 μm BaF_2_ sample cell. (*R*)-, (*S*)- and (±)-citronellal with purities of ≥90%, ≥96% and ≥95%, respectively, were purchased from Sigma-Aldrich. Both enantiomers have been measured as a neat liquid and in deuterated chloroform and acetonitrile solutions at a concentration of 0.3 M. The spectrum of the racemic mixture was used as a baseline for the VCD spectra. The conformational search has been performed using the RDkit and Macromodel software packages.^25^–^27^ In the two programs, 10 000 conformers were generated and subsequently optimized with UFF and MMFF, respectively.^28^,^29^ Further geometry optimizations and subsequent frequency and VCD calculations have been performed using Density Functional Theory (DFT) embedded in the Amsterdam Density Functional (ADF) software suite.^30^–^33^ The DFT calculations have been performed employing three different basis sets (DZP, TZP and TZ2P), four different functionals (BP86, OLYP, PBE, B3LYP), and two different dispersion corrections (DFT-D3 and DFT-D3-BJ), and using the COSMO solvation model for acetonitrile (EPS = 37.5, RAD = 2.76) and chloroform (EPS = 4.8, RAD = 3.17).^34^–^45^ The computed VA and VCD intensities have been convoluted with a Lorentzian function using a full width at half maximum of 8 cm^–1^. The computed frequencies have been scaled using the method developed by Shen *et al.*^46^ in order to obtain the correct overlaps between theory and experiment. Detailed descriptions of the employed genetic algorithm and the *k*-fold cross-validation procedure are given in the ESI Section S1.†

## 3 Results

### 3.1 Experimental results

The experimental VCD spectrum of dehydroquinidine has been reported previously.^24^ VCD spectra of the (*R*)- and (*S*)-enantiomers of neat citronellal given in ESI Fig. S2† show in general a good mirror image but there are a few minor differences, the additional band at 1052 cm^–1^ observed for (*R*)-citronellal being the most conspicuous one. Since this band and other differences are also observed in the VA spectra (see ESI Fig. S2†), we attribute them to the lower level of purity of (*R*)-citronellal (90%) with respect to (*S*)- and (*R*/*S*)-citronellal (96%). For comparison with the calculations we therefore only used the spectrum of (*S*)-citronellal and the spectrum of the racemic mixture for determining the baseline. Due to the high VA we were not able to measure the VCD spectra of neat citronellal accurately in the region between 1365–1471 cm^–1^. Since the calculations and experiments in solution show relevant bands in this region, the measured spectrum of citronellal in chloroform was used to compare with the calculations in that region. Such an approach is validated by ESI Fig. S3 and S4† that show no significant differences between the experimental and calculated spectra in neat and solution phases. For the carbonyl region no accurate VCD spectra could be measured due to its low vibrational dissimilarity factor.^47^

### 3.2 Gas-phase conformations

**Fig. 2:**
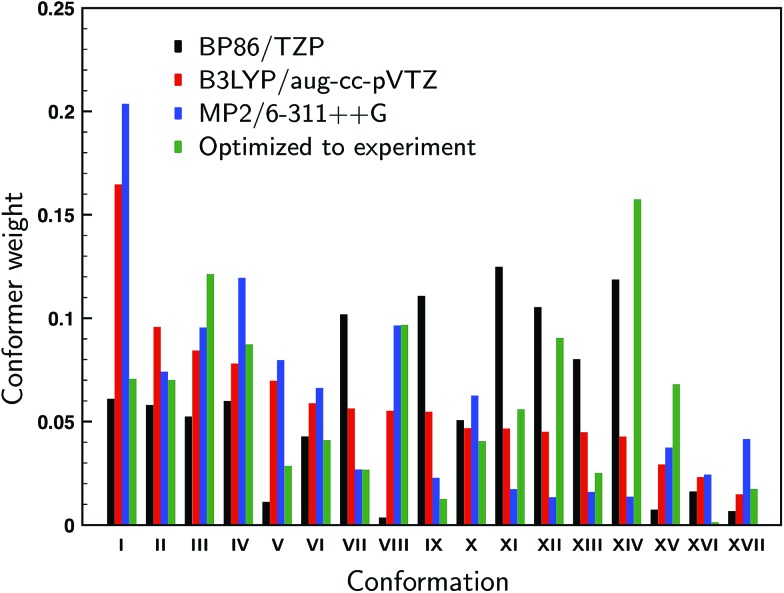
Computed Boltzmann weights at different levels of theory (black: BP86/TZP; red: B3LYP/aug-cc-pVTZ;^13^ blue: MP2/6-311++G^13^). The green weights derive from a fit of the experimental spectrum to the theoretically predicted VCD spectra using conformational weights derived from energies that have been allowed to change freely from their calculated values.

For the 17 conformations reported in the gas-phase study of Domingos *et al.*^13^ – of which two were not found experimentally – large differences are found in the relative computed energies and BWs for different levels of theory as can be concluded from Fig. 2 where we compare our calculations at the BP86/TZP level with the calculations at the B3LYP/aug-cc-pVTZ and MP2/6-311++G levels of theory presented in ref. 13. To quantify the agreement between the calculations and experiment we compute the overlap between experimental and predicted spectra using the SimVCD measure which can range from –1 for an exactly opposite spectrum to +1 for a perfectly matching one (see ESI Section S1†). Such a comparison leads for the BP86 and B3LYP calculations to similar SimVCD overlaps (0.6867 and 0.6840, respectively), while the – in principle superior – MP2 calculations yield a spectrum with a much lower SimVCD value (0.5496). The observation that the BP86 and B3LYP calculations give the same overlap despite the fact that different BWs are used is surprising, even more so because the spectra of the various conformations differ significantly.‡
‡The average SimVCD value between the spectra of the 17 conformations is only 0.03 with a standard deviation of 0.15. Interestingly, when we disregard the energies completely and just compute the arithmetic mean of the 17 spectra, a SimVCD value of 0.6828 is found. In other words, using the energies from either the BP86/TZP or B3LYP/aug-cc-pVTZ calculations to weigh the spectra is just as good as not weighting the spectra at all. Including dispersion or using a different exchange-correlation functional than BP86 (see ESI Fig. S5 and S6†) show that the spectra of the individual conformations vary largely between them, but do not vary much for different computational parameters, and thus show that this conclusion is not accidentally biased by the level of the calculation. It is therefore clear that there is a large error in the relative energies of the different conformations causing the BWs to have considerable less value than generally assumed.

In the gas-phase study of Domingos *et al.* it was concluded that conformations V and VIII (see ref. Domingos *et al.* for numbering and details of conformations^13^) were not present even though on the basis of their calculated energies one would have expected them to be present under the employed experimental conditions. One could therefore wonder whether these conformers are indeed also not present under room-temperature conditions, or whether they are present but during the cooling process converted to lower-energy conformations. Below we will introduce a novel approach in which the conformational population distribution is determined by fitting their calculated VCD spectra to the experimental spectrum using a genetic algorithm (see ESI Section S1†). Weights determined with this approach and using all 17 conformations are shown in Fig. 2. When we allow the energies of the individual conformations to vary freely from their computed energies we find identical optimized weights regardless of which of the three levels of theory was taken as the starting point. Looking at the weights we see that conformer VIII is predicted to contribute for 9.6% to the experimental spectrum, while the contribution of conformer V is much lower, but still 2.8%. When one of these two conformers is removed and the energies re-optimized a spectrum is obtained that shows a smaller overlap with the experiment: 0.8025 and 0.7893 when removing conformer V and VIII, respectively, against 0.8047 for the full conformational set. These results suggest that it is likely that conformer VIII is present under room-temperature liquid conditions, but that the presence of conformer V is considerably less certain.

### 3.3 Liquid-phase conformations

**Fig. 3:**
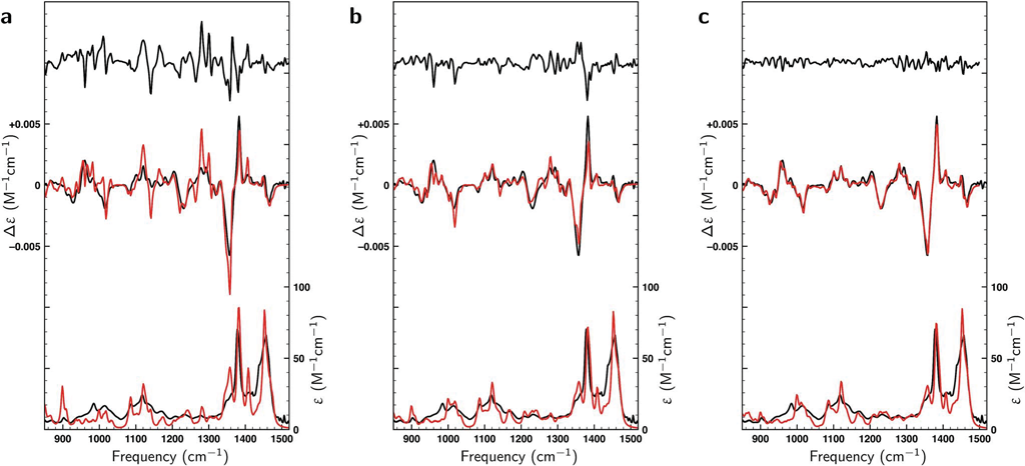
Comparison between experimental (black) and theoretically predicted (red) VA and VCD spectra of (*S*)-citronellal. (a) Comparison between the experimental spectrum with Boltzmann-weighted spectra using the 17 conformations reported in gas-phase studies^13^ with the upper trace giving the difference spectra between the two. (b) A similar comparison is made using the Boltzmann-weighted spectra of the 162 conformations resulting from the liquid-phase conformational search. (c) The right panel employs the same 162 conformations but uses weights as optimized with a genetic algorithm and a maximum energy modulation of 1 kcal mol^–1^.

In the liquid phase at room temperature citronellal is expected to be present in considerably more conformations than under the conditions of the gas-phase experiments in which molecules are cooled down rapidly into potential energy minima that are separated from other minima by relatively high barriers. We therefore performed an extended conformational search that was more adapted to the conditions of the liquid-phase experiments. Such a search resulted in 162 conformations with 70 within a 2 kcal mol^–1^ energy window at the BP86/TZP level of theory. Fig. 3 compares the experimental VA and VCD spectra with the BW-computed spectra based on the 17 conformations reported in the gas-phase studies and the 162 conformations in the liquid phase conformational search. As expected, the latter lead to a much better overlap with the experiment (SimVCD = 0.8012) than the former (SimVCD = 0.6867). In addition we see that many of the bands in the gas-phase prediction have an intensity that is too large and seem to be averaged out more in the experiment. These observations confirm that in the liquid phase many more conformations contribute to the spectra than in the gas-phase studies, and thus that the conformational space of citronellal is much more complex in real-life applications than might have appeared previously.

Above we concluded that the BWs of the gas-phase conformers were quite sensitive to the level of theory. The same and even more far-reaching conclusions can be drawn for the liquid-phase conformations. Here we find that not only the energies vary as much as found for the gas-phase conformations, but also that the actual number of conformations in particular energy windows differ strongly from one level of theory to the other (see Table S1†). The large number of low-energy conformations together with the uncertainty in those computed energies give rise to serious doubts on the accuracy of the calculated spectra. To assess this accuracy further we calculated the overlap between theoretical and experimental spectra in three ways. In the first approach, we take the arithmetic mean of the conformational spectra, which can be considered as the worst possible guess for the conformer weights. In the second approach we use the BWs associated with the BP86/TZP conformational energies, which in principle should lead to a better agreement. Finally, the best possible guess has been calculated by optimizing the BWs against the experimental spectrum using a genetic algorithm and a Δ*E*^max^ value of 1 kcal mol^–1^, the latter value implying that conformational energies were allowed to vary within ±1 kcal mol^–1^ from the energy values calculated at a particular level of theory (see ESI Section S1† for further details on the methodology and details of the employed genetic algorithm).

**Fig. 4:**
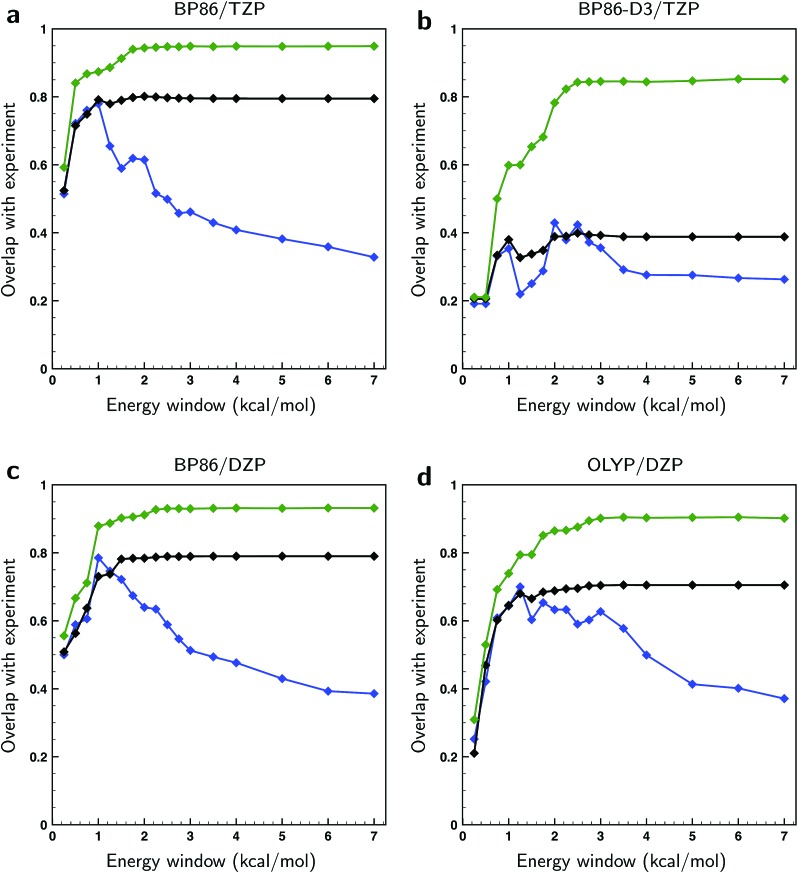
Overlaps between calculated and experimental VCD spectra of (*S*)-citronellal when taking conformers into account from differently-sized energy windows and using different computational parameters (see top of panels a–d). Blue traces have been computed using the arithmetic mean of conformational spectra, black traces using the BW spectra and green traces using weights of conformer spectra that have been optimized to the experiment with a genetic algorithm and a maximum energy variation Δ*E*^max^ of 1 kcal mol^–1^.

In Fig. 4 the overlaps are given for the three methods for different levels of theory. As expected, the overlaps calculated with optimized weights clearly give superior results and start to show significant differences with the other two methods already for relatively small energy windows, *i.e.*, taking only a relatively small number of lowest energy conformations into account. We find that the non-optimized BW spectra perform surprisingly poorly with respect to the arithmetic mean spectra when taking only low-energy conformations into account. Instead of outperforming the arithmetic mean, we see that up to a certain energy window size the two perform equally well. This observation can be rationalized if one assumes that the point at which the BW spectrum starts to outperform the arithmetic mean is closely related to the uncertainty in the energies. From Fig. 4 it can then be estimated that the energies computed at the BP86/TZP level have an uncertainty of about 1 kcal mol^–1^ while going to the DZP basis set results in a slightly higher value of 1.25 kcal mol^–1^. Remarkably, the calculations using a dispersion correction (BP86-D3/TZP) give by far the poorest results and lead to an estimated error of 2.5 kcal mol^–1^. This is probably due to the absence of explicit solvent molecules in the calculation to describe intermolecular dispersion interaction that compete with intra-molecular dispersion interactions. Moreover, from the large gap between the fitted and the BW spectra in Fig. 4b it can be concluded that the main reason for the poor comparison between experiment and calculations at the BP86-D3/TZP level is due to the error in the relative energies of the conformations. The spectra of the individual conformers computed with this dispersion correction, on the other hand, are found to be quite reasonable.

**Fig. 5:**
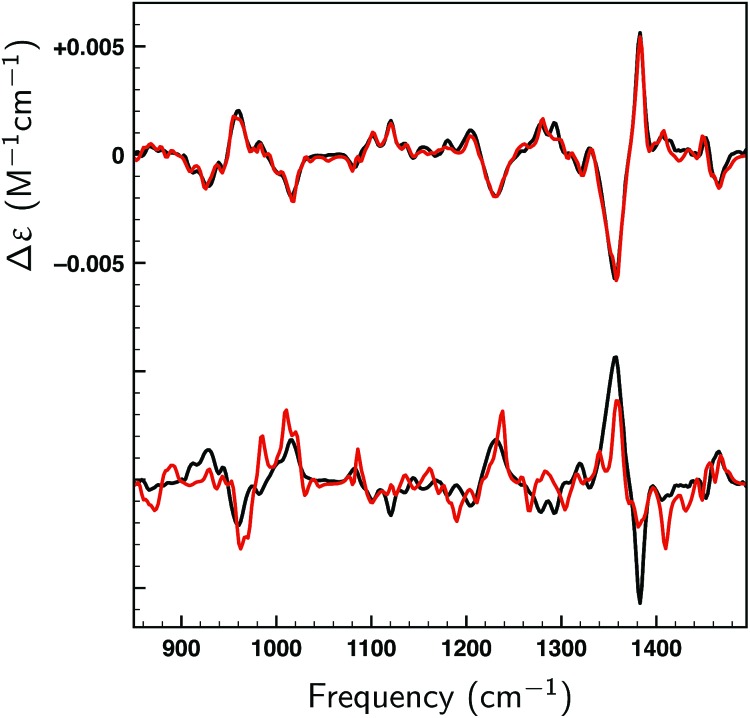
Fits (red) of the experimental VCD spectrum of (*S*) and (*R*)-citronellal (black) using the theoretically predicted spectra of the 162 conformers of (*S*)-citronellal found at the BP86/TZP level of theory using the genetic algorithm and a maximum energy modulation of 2 kcal mol^–1^. The top panel reports the fit to the (*S*)-enantiomer (SimVCD = 0.9678), the bottom panel the fit to the (*R*)-enantiomer (SimVCD = 0.5050).

In the present case there is no uncertainty about the enantiomeric assignment due to the large overlap between theory and experiment. For other flexible molecules and/or systems with even larger numbers of conformations, however, it could very well be that such an assignment is not so clear-cut. The question then might be whether it would be possible – due to the large error in the BWs and the large number of conformations – to simulate the wrong enantiomer. To investigate such a scenario we compare in Fig. 5 the experimentally recorded spectrum for the (*S*)- and (*R*)- enantiomer fitted with the spectra calculated for (*S*)-enantiomeric conformers. It should not come as much of a surprise that with 162 spectra of different conformations almost any spectrum can be fitted. Obviously, the fitted spectrum of the correct enantiomer looks nearly perfect, but the overlap of 0.51 found using the fit to the opposite enantiomer is also much higher than the threshold of 0.4 suggested previously to be sufficient to determine the absolute configuration.^2^,^48^ This clearly demonstrates that the spectrum of the individual conformers contain features of both enantiomers and that due to erroneous energies the incorrect enantiomer can in principle be simulated.

**Fig. 6:**
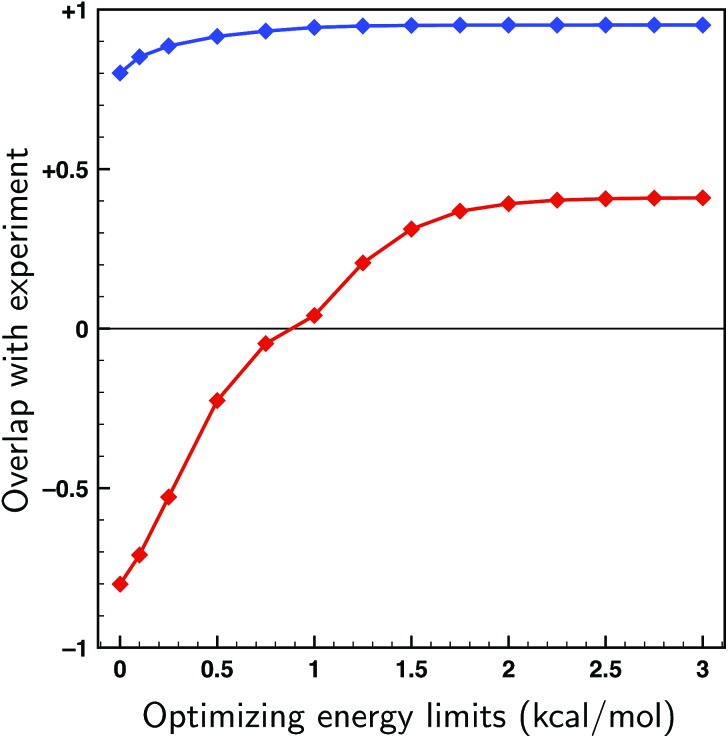
Fits to the experimental spectra of the two enantiomers of citronellal using the genetic algorithm and the 70 lowest energy conformers of citronellal found within 2 kcal mol^–1^ at a BP86/TZP level of theory as a function of the maximum energy modulation Δ*E*^max^. Blue: overlaps from the fit to the correct (*S*-)enantiomer. Red: overlaps from the fit to the incorrect (*R*-)enantiomer.

To asses whether allowing for uncertainties in the calculated energies might lead to an incorrect assignment of the absolute configuration, fits have been performed on both enantiomers of citronellal using the 70 lowest-energy conformers within a 2 kcal mol^–1^ energy window, which is double the expected uncertainty in energy (see Fig. 4). If there is a significant difference between the overlaps with the experimental spectra of two enantiomers, it is statistically unlikely that the wrong enantiomer is simulated. In cases where this difference is small, on the other hand, it is no longer possible to assign the absolute configuration at the used level of theory. In Fig. 6 the overlaps from the fits to both the correct and incorrect enantiomers are presented for different maximum energy modulations. This figure indicates that even for unrealistically large values of Δ*E*^max^ the difference between the fitted overlap with the correct and incorrect enantiomer stays around 0.5. If a maximum energy modulation is used close to the uncertainty we estimate from Fig. 4 (about 1 kcal mol^–1^) this difference is even 0.90. Until more research is done on different highly flexible molecules it is difficult to provide a definite limit on which overlap differences are still acceptable for not doubting an absolute configuration assignment using the approach presented here. However, for the moment a value of 0.4, similar to the value for the non-fitted overlap suggested by Polaverapu *et al.*,^2^,^48^ would seem reasonable.

**Fig. 7:**
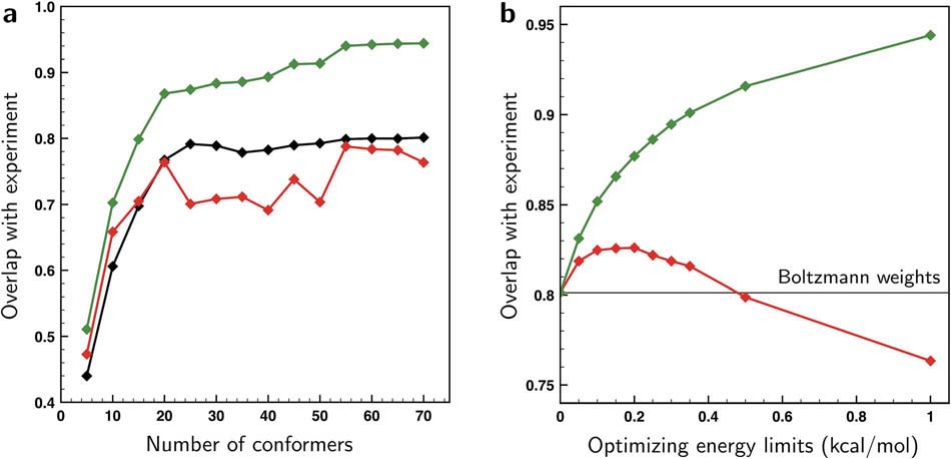
(a) Overlaps between the experimental VCD spectrum of (*S*)-citronellal and the combined average over the test sets in *k*-fold cross-validation runs taking different numbers of conformers into account (red trace). These overlaps are compared with overlaps computed using the BW spectra (black) and using weights of conformers that have been optimized to the experiment with a genetic algorithm and a maximum energy variation Δ*E*^max^ of 1 kcal mol^–1^ (green). (b) Overlaps between the experimental VCD spectrum of (*S*)-citronellal and the combined average over the test sets in *k*-fold cross-validation runs using the 70 lower-energy conformers found within 2 kcal mol^–1^ at a BP86/TZP level of theory for various values of Δ*E*^max^ (red trace). For comparison, the green trace depicts overlaps using weights of these 70 conformers optimized to the experiment with the employed genetic algorithm. The black line gives the overlap value when the Boltzmann weights are used based on the energies of conformers at the BP86/TZP level of theory.

From the excellent overlaps between theory and experiment observed in Fig. 4–6 one would in first instance be tempted to conclude that the present approach is able to provide an accurate description of the conformational distribution over all possible conformers considered (in this case 162). However, at the same time it is clear that the number of independent and distinguishing features in the measured VCD spectrum is considerably less than the number of conformers. To verify to what extent fitted weights can be used reliably from a statistical point of view to determine conformational weights, we have performed various *k*-fold cross-validation calculations taking different numbers of conformations into account and using different Δ*E*^max^ settings (see ESI Section 1.2†). The left panel of Fig. 7 shows such results for a Δ*E*^max^ value of 1 kcal mol^–1^. Taking up to 15–20 conformations into account, the test sets results show an overlap that is somewhere in between the BW spectrum and the optimized spectrum. This indicates that the results from the fit are indeed an improvement from the BWs. When more conformations are used, the overlap with the test sets results decreases and becomes less than the overlap with the BW spectrum. As these BWs are the starting point, this indicates that at this point overfitting starts to occur. We thus conclude that for the present case fitted energies and weights are reliable up to the 15–20 lower-energy conformations, but that for higher-energy conformations it is statistically not justified to make statements on population distributions. One further observation that needs to be taken into account is that for a small number of conformations the fit could be highly biased since it might be possible that not all important conformations are taken into account. A judicious choice of conformational space is therefore necessary.

As the Δ*E*^max^ parameter in the fitting procedure is an effective means to stop overfitting, it seems logical to look if the computed BWs can be improved by further tightening Δ*E*^max^ until the system is no longer overfitted. The effect of the Δ*E*^max^ parameter on the cross-validation results is depicted in the right panel of Fig. 7 where the energies of the 70 lower-energy conformations found within 2 kcal mol^–1^ at a BP86/TZP level of theory are optimized for different values of Δ*E*^max^. This panel shows that the overlap between test set and experiment initially increases up till a value of 0.2 kcal mol^–1^, and suggests that for larger values overfitting occurs. However, this does not mean that the fitted weights reflect the experimental situation since for small values of Δ*E*^max^ fitted energies will get stuck at the limit imposed by the Δ*E*^max^ value causing the calculated weight of those and other conformers not to be truly optimized. One therefore has to conclude that preventing overfitting by restricting Δ*E*^max^ is not recommended.

### 3.4 Dehydroquinidine

Thus far only citronellal has been considered for which – despite the large conformational heterogeneity – the comparison between experiment and theory is relatively easy. To test the performance of the algorithm for a ‘worst-case’ system, we have also analysed the VA and VCD spectra of dehydroquinidine. As explained in the introduction, the comparison between theory and experiment is much more complex for this molecule which is partly due to the quality of the experimental spectrum, but also because of the large-amplitude motions involving the hydroxy group (see Fig. 1) and the flexibility of the molecule in general. Taking the experimental and computed spectra of the 30 conformations and their enantiomers from ref. 24, the genetic algorithm was used to optimize the energies with a Δ*E*^max^ of 1 kcal mol^–1^ to the experimental spectrum.

**Fig. 8:**
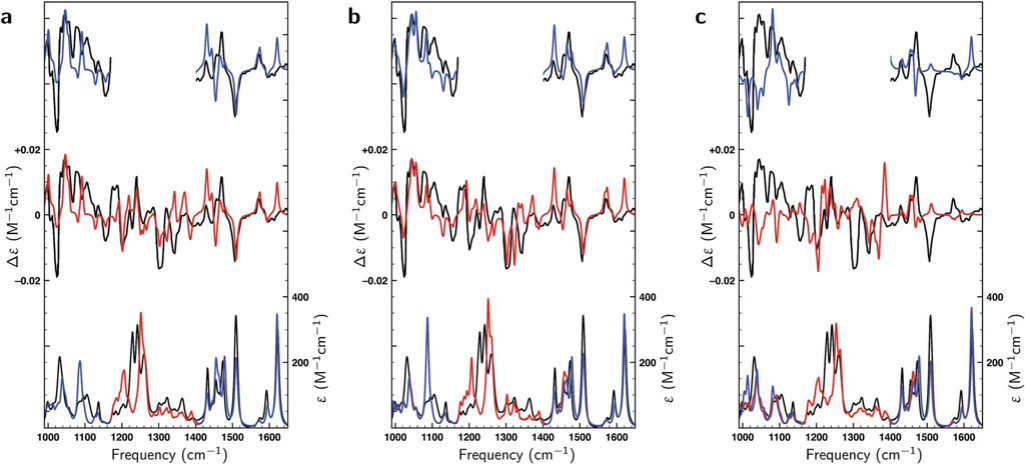
Comparison of experimental VA and VCD spectra (black) and calculated spectra of dehydroquinidine for the full frequency region (red) and spectra in which the 1200–1400 cm^–1^ region has been excluded (blue). Panel (a) depicts spectra using Boltzmann weights obtained at the BP86/TZP level. In panel (b) experimental spectra have been fitted to theoretical spectra with a Δ*E*^max^ = 1 kcal mol^–1^ energy modulation, while in panel (c) fits have been made to the calculated spectra of the opposite enantiomer.

Fig. 8a displays VA and VCD spectra using the Boltzmann weights as calculated from the energies of the various conformations at the BP86/TZP level of theory. The VA spectrum agrees quite well with experiment except in the 1200–1400 cm^–1^ region where clearly some bands are missing or predicted to have a very different intensity. This is reflected in the BW VCD spectrum which shows a significantly poorer agreement in this region leading to a SimVCD value of 0.3319. Since we concluded in ref. 24 that the 1200–1400 cm^–1^ region is dominantly affected by structural dynamics that cannot be reproduced by ‘static’ calculations such as the present ones, we also calculated the overlap leaving out this region, but even that only increased the overlap to 0.3623. Both values are below the threshold of 0.4 normally taken to assign confidently the absolute configuration.

In Fig. 8b a similar comparison is made but now using weights obtained from fitting either the full spectrum (red trace) or the spectrum with exclusion of the 1200–1400 cm^–1^ region (blue trace). Fits to the full spectrum clearly improve the overlap between experiment and theory (SimVCD = 0.4160), but they still fail to lead to significant improvements of the 1200–1400 cm^–1^ region. This is an important observation as we had concluded previously that the reason for the poor overlap in this region arises from the physical properties of the molecule and not from an inadequate description of conformational energies. This is encouraging and allows us to conclude tentatively that our fitting approach does not eliminate the effects of physical phenomena intrinsic to a molecule. Accepting that the 1200–1400 cm^–1^ region cannot be described correctly if structural dynamics are not explicitly taken into account, we have also performed fits excluding this region which lead to an overlap of 0.5324.

It is important to notice that the fact that both overlaps are larger than the threshold normally taken for a secure assignment of the absolute configuration (SimVCD > 0.4) should be taken with caution. Firstly, the threshold of 0.4 is based on spectra constructed with Boltzmann weights as calculated, that is, not fitted. Such spectra are always inferior to spectra in which these weights have been fitted to the experimental spectrum. Secondly, given enough conformers with significantly different spectra, almost any spectrum can be fitted as illustrated by the fits to the two enantiomers of citronellal (Fig. 5). An unambiguous criterion for assessing the reliability of a stereochemical assignment is therefore to determine to what extent the same fitting procedure is capable to fit the experimental spectrum with the spectra calculated for the different enantiomers. In Fig. 8c we show such fits for dehydroquinidine. It is highly gratifying to find also in this ‘worst-case’ example that such fits lead to poor overlaps of –0.0575 and 0.0043 for the full and trimmed spectrum, respectively. These values are far worse than for the correct assignment and leave no doubt on the stereochemical assignment of the molecule.

## 4 Conclusions

In the present study we have introduced a novel approach based on VCD spectroscopy to elucidate the conformational heterogeneity of flexible molecules, and to determine the consequences of such a heterogeneity for the assignment of the absolute configuration of chiral molecules. This is particularly important when considering such molecules under real-life conditions, as has become immediately clear from a comparison of the results of rotational gas-phase spectroscopic studies performed previously with the liquid-phase room-temperature VCD studies presented here. Firstly and not unexpected, this comparison showed that many more conformations are present under the latter conditions. Secondly, however, they also indicated that at least one of the two conformations expected to be present under gas-phase conditions but not observed, is indeed there under native conditions.

The standard approach employed so far has been to use Boltzmann weights to construct a predicted VCD spectrum on the basis of the VCD spectra of the individual conformers. However, our studies have shown that the uncertainty in the energy of the different conformations has dramatic consequences on the appearance of the VCD spectrum and thereby on the reliability of an absolute configuration assignment. In fact, under certain conditions it appears that a better agreement can be obtained between experiment and theory if a ‘wisdom of the crowd’ approach is used, that is, simply taking the arithmetic mean of spectra. We have shown that taking this energy uncertainty explicitly into account using a genetic algorithm based fitting procedure that fits the experimental spectrum to the calculated spectra leads to a significant improvement while at the same time giving a quantitative measure of the probability that an incorrect assignment of the absolute configuration occurs. Importantly, the comparison between the performance of the ‘wisdom of the crowd’ approach and the regular BWs has been argued to provide a direct estimate of the energy uncertainty and can thus also be quantified by independent means. *K*-fold cross-validation studies have demonstrated to what extent the genetic algorithm can provide statistically justified statements on the relative contributions of the various conformations. For citronellal we find that up to 15–20 conformations can reliably be treated, but this will clearly change from case to case and depend on the variation within the conformer spectra, the complexity of the experimental spectrum and the uncertainty in the computed energies.

Finally, the algorithm can be used to asses the quality of the computed BWs and spectra which in turn allows to determine whether differences between the experimental and predicted spectrum are due to errors in the spectra or in the calculated energies. A large error in the energies indicates that better suited computational parameters should be used. A large error in the spectra, on the other hand, indicates that a significant part of the pertaining chemistry is missing in the calculations. This can either be the absence of important conformations, solvents effects or the effects of important large-amplitude motions as has been illustrated by the ‘worst-case’ example of dehydroquinidine.^24^,^49^,^50^ To assess the full applicability range of the here presented methodology clearly requires more studies ‘in the field’. The present study, however, gives confidence in its robustness and indicates that it will be useful in various areas where conformational heterogeneity is important.

## Conflicts of interest

There are no conflicts to declare.

## Supplementary Material

Supplementary information
