# Supplementary material for: Taming conformational heterogeneity in and with vibrational circular dichroism spectroscopy
Source: Chem Sci. 2019 Jul 9;10(33):7680–9. doi: 10.1039/c9sc02866h (PMC6844231; doi:10.1039/c9sc02866h)
Supplement: Supplementary file 1 [file SC-010-C9SC02866H-s001.pdf]

Electronic Supplementary Material (ESI) for Chemical Science.
This journal is © The Royal Society of Chemistry 2019

Supporting Information

Taming conformational heterogeneity in and with Vibrational Circular Dichroism spectroscopy

M.A.J. Koenis[a],Y. Xia[a], S.R. Domingos[b], L. Visscher[c], W.J. Buma*[a,d], V.P. Nicu*[a,e]

[a]Van 't Hoff Institute for Molecular Sciences, University of Amsterdam, Science Park 904, 1098 XH Amsterdam, The Netherlands

[b]Deutsches Elektronen-Synchrotron DESY, Notkestrae 85, 22607 Hamburg, Germany

[c]Amsterdam Center for Multiscale Modeling, Division Theoretical Chemistry, Faculty of Sciences, Vrije Universiteit Amsterdam, De Boelelaan 1083, 1081 HV Amsterdam, The Netherlands

[d]Radboud University, Institute for Molecules and Materials, FELIX Laboratory, Toernooiveld 7c, 6525 ED Nijmegen, The Netherlands

[e]Lucian Blaga University of Sibiu, Faculty of Agricultural Sciences, Food Industry and Environmental Protection, 7-9 Ioan Ratiu Street, 550012 Sibiu, Romania

*email: w.j.buma@uva.com, v.p.nicu@gmail.com

1 Fitting experimental VCD spectra with a genetic algorithm

The starting point of our approach is that we do not take the calculated conformational energies as fixed and use Boltzmann weights based on these energies. Instead, we fit the experimental spectrum with the VCD spectra predicted for the various conformations, albeit that their weights are restricted by limits we impose on how much the energy of a particular conformation is allowed to vary. Genetic algorithms are frequently used and very suitable to optimize such a set of energies or conformer weights to experiments. This type of machine learning has been used successfully in gas-phase rotational spectroscopy to fit experimental spectra [1,2] but also in many other applications involving spectroscopic techniques. [3–5] Genetic algorithms mimic the process of evolution where a population of individual solutions are continuously modified (mutated, mixed, etc.) while keeping only the 'fittest' solutions for the next generation. Over many successive generations the population then evolves in such a way that it functions optimally under the specified conditions.

A schematic representation of the genetic algorithm employed in the present studies is shown in Scheme S1. Initially, a first energy set is created based on the energies as calculated with DFT. The other energy sets are mutated versions of this set with random mutations added to each energy. The maximum mutation is here the maximum allowed energy variation ΔE^{max}, which is given as an input variable and prevents overfitting to unrealistic energy values. In total 25 sets of energies are initialized which are then converted to 25 sets of Boltzmann weights and 25 computed Boltzmann weighted spectra.

The next step is to identify the '*fittest*' sets to be used for generating a next generation of sets. In order to do so we compute the overlap of the theoretically predicted spectra with the experimental one using the SimVCD measure which can range from –1 for an exactly opposite spectrum to +1 for a perfectly matching one: [6]

$$SimVCD = \frac{I_{ce}}{I_{cc} + I_{ee} - |I_{ce}|} \tag{1}$$

where

$$I_{ij} = \int F_i(v) F_j(v) dv \tag{2}$$

$F_e(v)$ and $F_c(v)$ being the experimental and computed intensities at frequencies v respectively. In principle, any similarity measure can be used but we have chosen here for SimVCD because of its low computational cost (it must be computed millions of times) and because of its smooth transition between positive and negative overlaps.

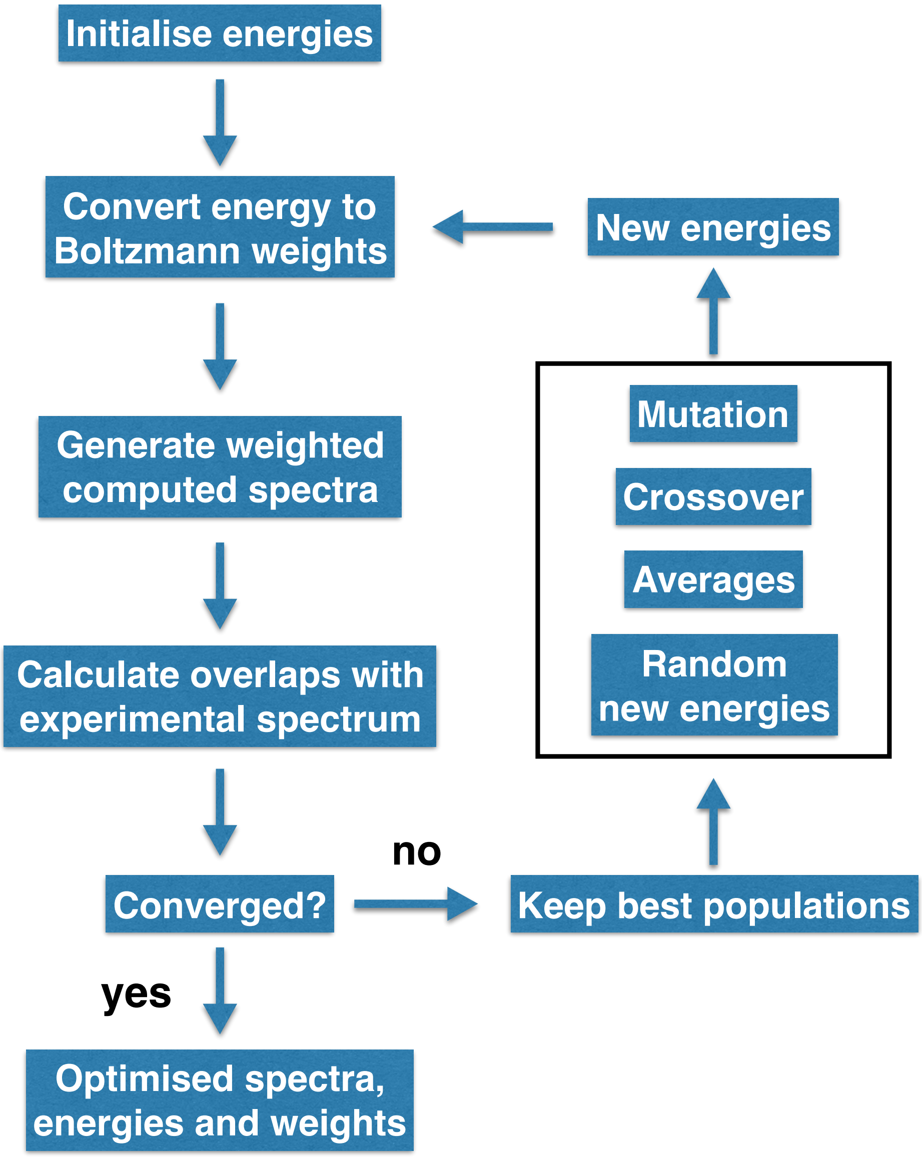

Figure S1: Schematic representation of the genetic algorithm developed to optimize the relative computed energies of the conformations to an experimentally recorded VCD spectrum.

The next step in the genetic algorithm is to converge to the optimal fit. In our case we considered the system as being converged when the overlap with the experimental spectrum did not improve over 2000 generations. If the system is not converged, the three sets of populations with the best overlap are used as a basis to generate the next set of populations. Here two regimes can be identified: exploration and optimization. The advantage of this separation is that it allows for a faster optimization while maintaining a broad exploration of the parameter space. In the exploration regime the focus is on exploring the large parameter space to identify possible overlap maxima. The top three population sets in this regime are forced to be significantly different from each other and new populations are formed with relatively large mutations, averages and many random new sets. Once the algorithm starts to converge towards a specific maximum it switches to the optimization regime. During this phase much smaller mutations are used to create new sets of populations, crossover sets are no longer based on random weights but on the weights of the other population sets, and only a few random population sets are employed. The final random spectra are purely used to reduce the probability that the optimum that has been found is not the global maximum.

There are several implications that should be taken into account when using a genetic algorithm in order to optimize the bonding energies with respect to experiment. First, the higher the number of conformations the more difficult it will become to optimize the structure. This is simply because the change of 'quasi' randomly finding a better set of energies becomes more difficult when more parameters are allowed to be changed. The algorithm presented here had no difficulties optimizing the energies of up to 100 conformations. For systems with more conformations multiple runs were required in order to assure the optimal energies were found. We estimate that at 150 conformations there is only a 5-10% change for the system to optimize correctly and that at about 200 conformations the algorithm is highly unlikely to find a optimum. In principle this can be countered by increasing the number of sets in the population drastically. However, this will also drastically increase the time needed for the optimization. Secondly, the optimization will only work when the spectra of conformations within the given energy window are significantly different. When two conformer spectra are highly similar the two conformations cannot be separated and their relative energies can not be optimized. Still, the sum of such conformations can provide some useful information. Lastly, it should be noted that the genetic algorithm is by no means crucial for the fitting of the energies to an experimental spectrum. In principle any global optimization scheme for a function of a few tens to hundreds of parameters can be used.

1.1 Details of the used genetic algorithm

At the start of the optimization, in the "Exploration" regime, the best three energy sets that are taken to the next generation must be significantly different from each other (at least 0.01 total difference in weights). Then the top three energy sets are mutated 2 times by sets of

random numbers (resulting in 6 sets), the first mutation has a maximum of 0.01 times the maximum allowed energy difference, the second mutation limit is 2 times larger. When a mutation brings a energy outside its allowed region the energy is reassigned as at a random value inside this range, allowing for extra exploration of the parameter fields. 4 more sets are made using averages of the top three; 3 averaging only pairs and 1 averaging all three. the remaining 15 populations are totally random new sets of energies with the only limitation that the energies of the conformations should lie within the energy range specified by the user in the beginning. Once the genetic algorithm starts to converge towards a specific maxima (we used as criteria less than 0.0005 difference in SimVCD value in 500 generations) the algorithm is switched to the optimization regime which focusses on optimizing towards the found maxima.

In the "optimization" regime the minimal allowed difference within the top three sets is reduced to 0.001 total difference in weights allowing for similar populations to taken into the mixing step. Then the top three energy sets are mutated 4 times with maximum random energy mutations of 0.001, 0.002, 0.005 and 0.01. When a mutation brings the energy outside its allowed region it was now brought back to the correct region. Next cross-over energy sets were generated of the top 3 energy sets with the odd and even energies from different sets were combined. Lastly only 4 totally new random energies were created. This step continued until 2000 new generations did not improve the overlap with the experimental spectrum or was stopped after 100000 steps. At that point only insignificant changes to the energies and corresponding simVCD occur.

1.2 K-fold cross-validation

To quantify the prediction capabilities of the genetic algorithm with regards to the conformer weights a specialized cross-validation was performed. The applied method is close to the K-fold cross-validation approach[7] in which the data that are modelled are split K times in a test and training set. The training set is used to fit the model which is then validated using the test set. This K-fold cross-validation method usually selects the data points for the test and training sets randomly, but in the present case this is not possible since the data points in the experimental spectrum are correlated (consider, for example, the data points of one particular vibrational band). We therefore used instead 10% of the frequency range which corresponds to 65 cm^{-1} and contains 2-3 spectral features. Since the training results depend strongly on the exact cuts for the test spectrum, we performed 100 validation runs (ten times that of the standard K-fold cross validation method) during which the location of the test set was chosen quasi-randomly (during the first 10 runs the test set was chosen such that the entire spectrum was covered at least once by the test sets). Additionally, a buffer section -set equal to the FWHM used to broaden the computed stick spectrum with a Lorentzian- was defined that was not used in either the training nor the test set. This buffer ensured that the edges of the test spectrum did not correlate with the neighbouring edges of the training spectrum. A final issue that needs to be taken into account with this type of data is that the errors between the

model and experiment are not distributed uniformly over the dataset. Instead, there is a clear structure with the error increasing or decreasing roughly with the spectral absorption intensity. This means that different parts of the spectrum have different deviations between experiment and simulation and thus different SimVCD values. Therefore only SimVCD values of the same spectral range can be compared with each other and only the combined average over the test sets can be compared to the simulation of the full spectrum.

The accuracy of the fitted energies and Boltzmann weights depends on several things: the variance of the fitted weights and whether the data is being overfitted. The variance of the fitted weights can be assessed by looking at the results from the 100 cross-validation runs and is affected by both the shape of the spectrum of the conformer and on its uniqueness. Furthermore, if a conformer spectrum stands out in just a small section of the spectrum its importance will drop significantly when that section is in the test or buffer section of the spectrum. A good estimation of the error in the fitted weights is de maximum difference within the 100 validation runs. This takes both above mentioned sources of variation into account and has a build-in safety margin since the fit of the full spectrum will always be more accurate than the <90% that is fitted in the training sets. Further, in this type of fitting procedure there is a high risk of overfitting since both the model and the data that is being fitted contain errors. In order to prevent overfitting we looked at the SimVCD between the average of the test spectra and the experimental spectrum. If this SimVCD value is lower than at the start of the optimization - the SimVCD with the Boltzmann weighted spectrum - it means that training sets have been over fitted and that the resulting conformer weights are probably no improvement any more over the computed Boltzmann weights. Both these restrictions impose large but realistic limitations on the trustworthiness of the fitted Boltzmann weights. However, regardless of the believability of the weights, one should keep in mind that the resulting spectra is always within the error margin of the calculations as long as the maximum allowed energy change is within the accuracy of the applied computational method and thus always demonstrate the best possible scenario.

2 Supporting Information Figures and Tables

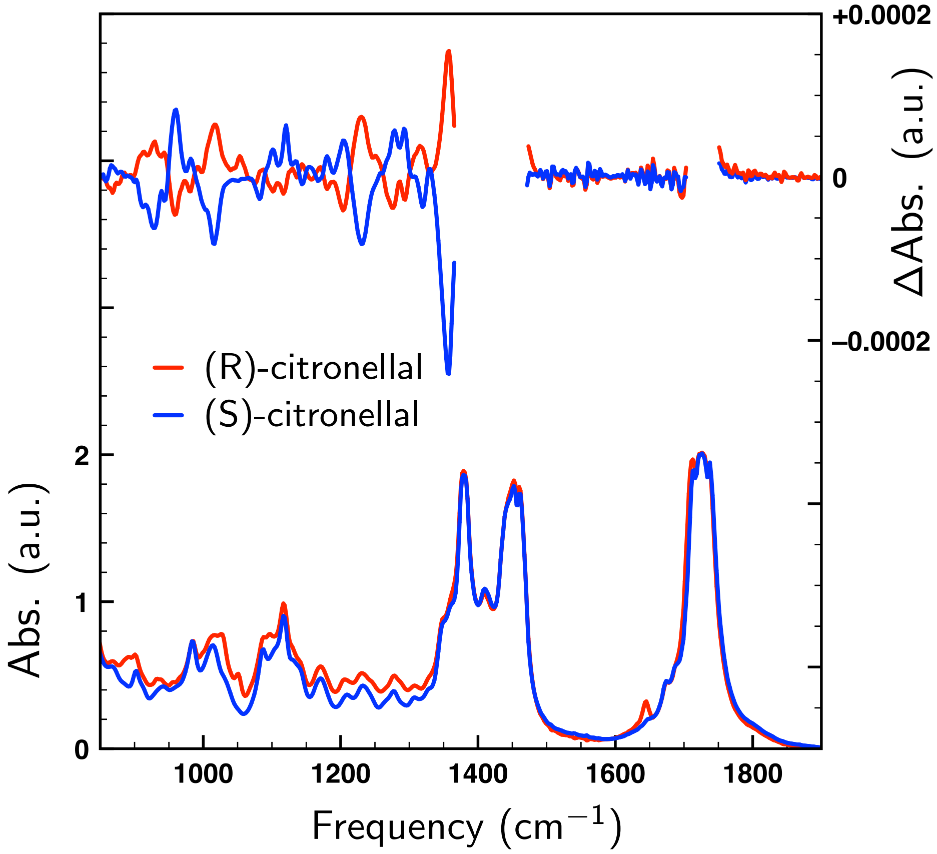

Figure S2: Experimental VA and VCD spectra of the (R)- and (S)-enantiomers of neat citronellal using a 75 μm path length.

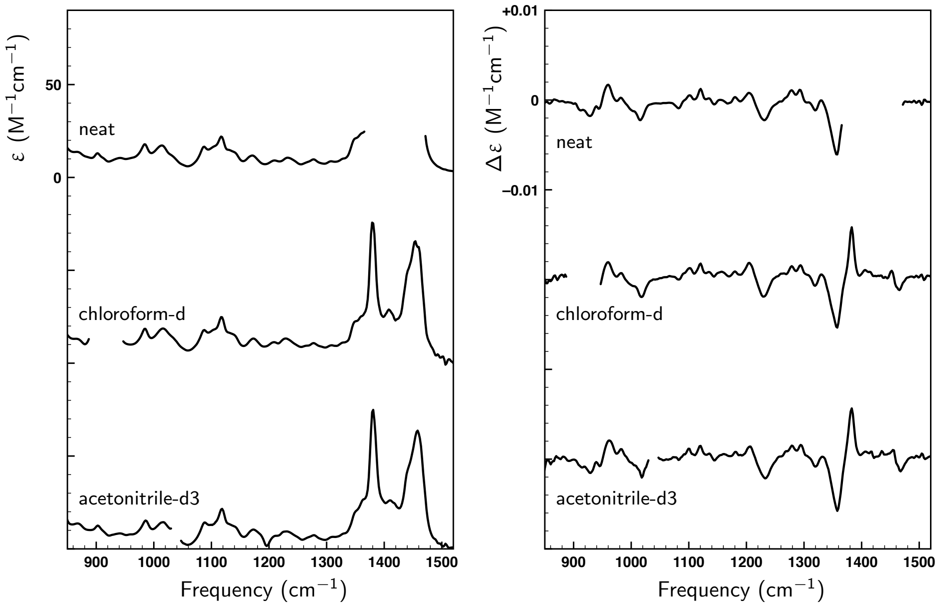

Figure S3: Comparison of the experimental VA and VCD spectra of the (S)-enantiomer of neat and dissolved citronellal in deuterated chloroform and acetonitrile. A concentration of 0.3M was used in combination with a 75 μm path length.

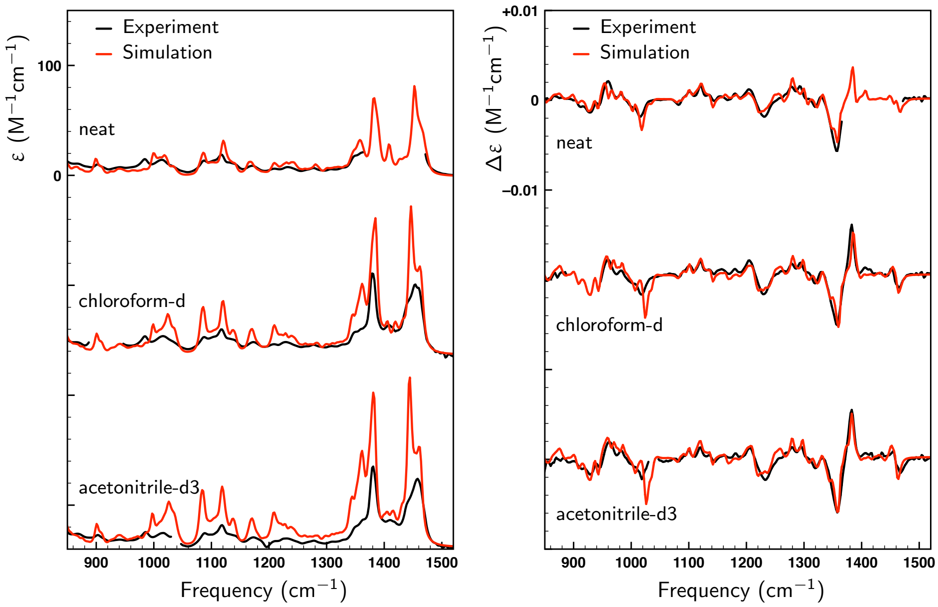

Figure S4: Comparison between the computed and experimental VA and VCD spectra of the (S)-enantiomer of neat and dissolved citronellal in deuterated chloroform and acetonitrile. A concentration of 0.3M was used in combination with a 75 μm path length.

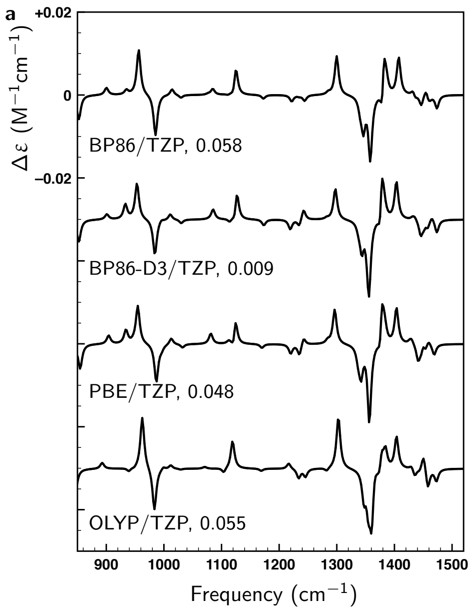

Figure S5: Comparison between the computed VCD spectra of (S)-citronellal computed at a BP86/TZP, BP86-D3/TZP, PBE/TZP and OLYP/TZP level of theory. For comparison also their relative Boltzmann weights are given. Shown are calculations for conformers 8 (a) and (9), the two lowest-energy conformations at the BP86/TZP level of theory.

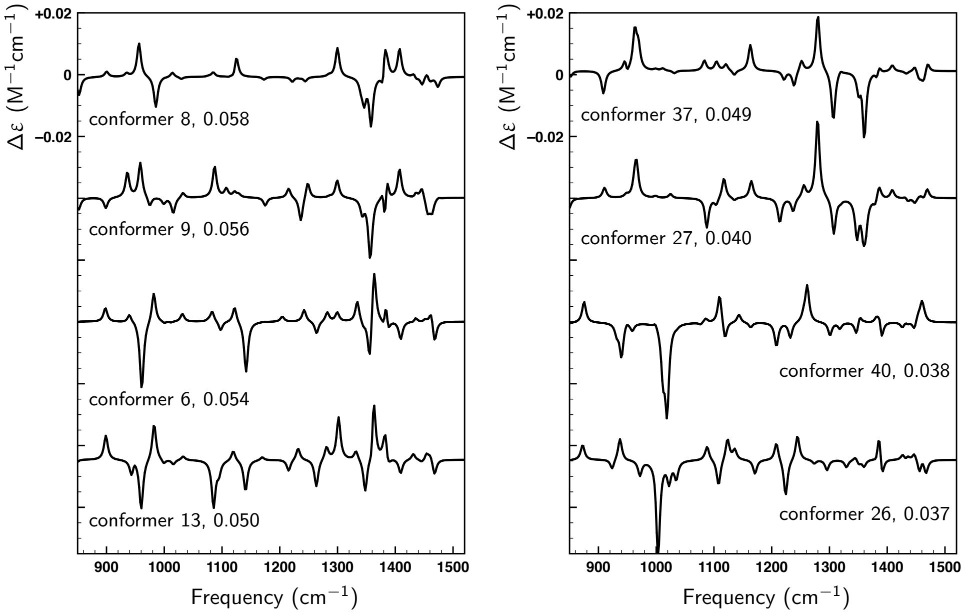

Figure S6: Comparison between computed VCD spectra of the 8 lowest-energy conformations of (S)-citronellal at the BP86/TZP level of theory with their relative Boltzmann weights.

	Energy window (kcal/mol)			
Parameters	1	2	3	8+
BP86/TZP	24	70	107	162
BP86/TZ2P	25	69	107	158
BP86/DZP	29	66	110	158
OLYP/TZP	14	49	84	140
OLYP/TZ2P	15	48	76	135
OLYP/DZP	17	38	71	134
B3LYP/TZP	21	64	107	160
PBE/TZP	27	77	114	162
BP86-D3/TZP	15	45	117	162
BP86-D3-BJ/TZP	12	64	107	155

Table S1: Number of structures within given energy windows from their respective lowest energy conformation when using different basis sets and functional. The conformers found from the conformational search have been optimized at different levels of theory. 10-25% of the conformations converged to the same structure and was removed. This depends strongly on the level of theory and leads to large differences in numbers of found conformations.

References

[1] J. A. Hageman, R. Wehrens, R. de Gelder, W. Leo Meerts and L. M. C. Buydens, *J. Chem. Phys.*, 2000, **113**, 7955–7962.

[2] W. L. Meerts and M. Schmitt, *Phys. Scr.*, 2005, **73**, C47.

[3] G. J. Metzger, M. Patel and X. Hu, *J. Magn. Reson., Ser. B*, 1996, **110**, 316.

[4] H. Xie, J. Zhao, Q. Wang, Y. Sui, J. Wang, X. Yang, X. Zhang and C. Liang, *Sci. Rep.*, 2015, **5**, 10930.

[5] M. Yin, S. Tang and M. Tong, *Anal. Methods*, 2016, **8**, 2794–2798.

[6] J. Shen, C. Zhu, S. Reiling and R. Vaz, *Spectrochim. Acta Part A: Mol. Biomol. Spectrosc.*, 2010, **76**, 418 – 422.

[7] M. Stone, *J. Royal Stat. Soc., Ser. B*, 1974, 111–147.
